# Canine Descemet Stripping Endothelial Keratoplasty with a Tissue Insertion Device: Technique and Long-Term Outcome

**DOI:** 10.1155/2023/7497643

**Published:** 2023-12-21

**Authors:** Conan Y. Chen, Steven J. Solar, Daniel S. Lewis, Kali Barnes, Batya G. Wiener, Satya Baliga, Eric Chiang, Tim E. Askew, Allen O. Eghrari, Micki D. Armour

**Affiliations:** ^1^Robert Wood Johnson University Hospital, New Brunswick, NJ, USA; ^2^Department of Biomedical Engineering, Johns Hopkins University, Baltimore, MD, USA; ^3^LuckyVision, Washington, DC, USA; ^4^Johns Hopkins Medicine, Baltimore, MD, USA; ^5^Armour Veterinary Ophthalmology, Washington, DC, USA

## Abstract

*Introduction*. We describe a case of canine Descemet's stripping endothelial keratoplasty (DSEK) using an open-source canine tissue delivery device. *Case Presentation*. We follow the four-year outcomes of a 1.5-year-old Tibetan Terrier who presented with difficulty seeing, diffuse corneal edema, and central corneal thickness of 1400 microns in the left eye. To perform DSEK, a polycarbonate carrier and insertion device was designed for canine corneas that measure up to 15 mm in diameter. The tissue was loaded into the inserter prior to surgery with the endothelium facing inwards and the stroma facing the cartridge wall. From the cartridge, the graft was pulled into the eye using microforceps and an anterior chamber maintainer. We assessed preoperative endothelial cell count, postoperative corneal clearance, and graft adhesion. The donor was a two-year-old Airedale Terrier who died one day prior to surgery, with endothelial cell density of 3149 cells/mm^2^. One week after DSEK, the cornea began to clear, and pachymetry of the donor and graft total was 1410 microns. This improved to 800 microns at 4 months and continued improving in its clarity at the last postoperative visit 4 years after surgery. *Discussion*. We demonstrate the feasibility of conducting canine endothelial keratoplasty with a specially designed tissue delivery device and the potential of long-term corneal clearance after DSEK in canine eyes.

## 1. Introduction

Corneal endothelial dystrophy (CED) is a disabling disorder of the cornea characterized by endothelial cell decompensation, formation of bullae, and ulcerative keratitis.

To date, the treatment of canine corneal dystrophies has generally included various approaches including superficial keratectomy and use of conjunctival flaps [1–3]. Recently, the concept of Descemet's stripping endothelial keratoplasty (DSEK) was introduced for canine CED [4, 5], which can result in prolonged optical clarity by directly addressing the underlying endothelial failure. This procedure involves removal of the recipient Descemet's membrane and replacement with a posterior corneal lamellar graft with viable endothelium.

One challenge associated with canine endothelial keratoplasty (EK) procedures is a lack of equipment designed to accommodate or transport a larger tissue graft, as canine elliptical corneas are typically 15 to 20 mm compared to 10 to 11 mm in humans [3]. Protection of the endothelial cells is imperative to successful incorporation of the graft, and transferring the graft from the corneoscleral rim through the wound and into the anterior chamber can result in endothelial cell loss. To address this gap in care, a specialized device was designed to facilitate endothelial keratoplasty in canines suffering from CED.

In addition, there is a need for long-term data regarding outcomes of canine EK. Previous reports of canine DSEK have demonstrated good outcomes at 3 and 12 months after surgery [2, 3], but it is unclear whether grafts can remain clear beyond a year. Here, we describe the treatment of a canine eye with DSEK, demonstrate the feasibility of corneal clearance over 4 years after surgery, present the use of a dedicated canine tissue inserter for endothelial keratoplasty, and share the design files openly for further replication or iteration.

## 2. Case Presentation

### 2.1. Preoperative Evaluation

**Figure 1:**
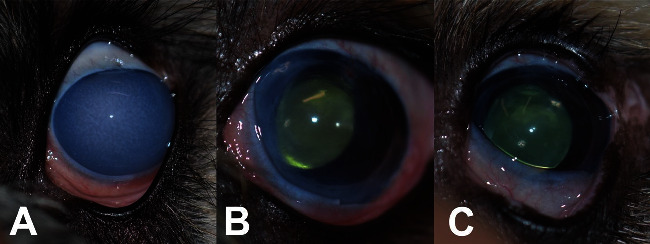
Photographs of an eye affected by canine endothelial dystrophy before and after Descemet's stripping endothelial keratoplasty (DSEK) in a 16-month-old Tibetan Terrier. Preoperatively (A), diffuse edema had resulted in complete opacification of the cornea, with no visibility of anterior chamber structures. Corneal pachymetry measured 1400 microns centrally. Twelve days after surgery (B), the cornea began to clear, and the pupil became visible. Pachymetry improved to 800 microns at 4 months. At 40 months (C), the graft remained clear and opacification in the peripheral cornea continued to resolve. The iris and lens are now clearly visible.

In this case report, a 1.5-year-old Tibetan Terrier was referred for corneal edema which developed after phacoemulsification. The left cornea became diffusely edematous and did not improve despite use of hypertonic saline. The right eye was unremarkable. Pachymetry was 1400 microns, and the edema reached a level of diffuse opacification, as visible in Figure 1.

Upon presentation, we performed a complete ophthalmic examination including slit-lamp biomicroscopy, binocular indirect ophthalmoscopy, rebound tonometry, and fluorescein stain. Notably, the view did not allow adequate visualization of the retina through indirect ophthalmoscopy. Ultrasonic biomicroscopy was performed with the Accutome UBM Plus device, through which a central corneal thickness of 1400 microns was measured. There was no epithelial defect visualized with fluorescein.

Corneal edema was assessed according to a grading scheme as described by Thomasy et al. and assessed to be grade 4 out of 4 (severe edema) [6]. Indeed, the left cornea demonstrated severe edema throughout and did not improve with hypertonic saline nor a topical nonsteroidal anti-inflammatory.

Informed client consent was obtained prior to undergoing endothelial keratoplasty. The procedure was performed following the guidelines of the Association for Research in Vision and Ophthalmology for use of animals in research.

### 2.2. Development and Use of Corneal Graft Insertion Device

While we and others have previously reported a canine tissue insertion technique that involves directly pulling or pushing tissue into the anterior chamber through the wound, human studies have suggested that an insertion device that bypasses the risk of trauma from wound entry can offer improved outcomes [7]. As there are currently no devices to facilitate the corneal transplant with accommodations for the size and shape of the canine corneal graft, we focused on designing a solution to address this issue.

**Figure 2:**
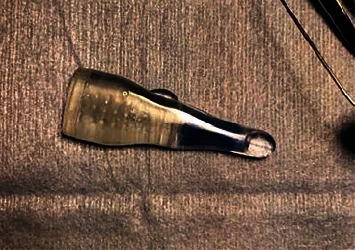
The graft, stained with trypan blue to assist with visualization, is loaded into the inserter prior to surgery and pulled close to the tip to assist with its delivery using a pull-through method.

Our design requirements and manufacturing process are described in Supplemental Protocol 1. We required a device that could accommodate the size of a 15 mm graft but still be able to fit within a 6 mm incision. SolidWorks software was used to design the device, and final devices were injection-molded using medical-grade polycarbonate through Xcentric Mold and Engineering (Clinton Twp, MI). Watertight caps were 3D printed using FormLabs 3 (Baltimore, MD). We describe this device as the “Luna Inserter” and openly share the design files free of charge for iteration. A depiction of the inserter, preloaded with the graft stained with trypan blue, is included in Figure 2.

### 2.3. Donor Tissue Preparation

Identification and procurement of viable, healthy donor tissue is essential for the success of the procedure. Our approach to planning, procurement, and preparation of donor tissue is outlined in Supplemental Protocol 2. This includes identifying donors, determining tissue eligibility standards, recovering tissue, assessing tissue quality, and cutting DSEK grafts.

Due to the diffuse corneal edema, we appreciated that visualization would be limited and stained the corneal stroma of the graft with trypan blue to assist with visualization. The graft was then preloaded into the anterior aspect of the Luna Inserter with assistance of 23 G microforceps. The cartridge was filled with Optisol. The inserter was then flipped for entry into the anterior chamber.

Donor tissue was acquired from a 2-year-old Airedale Terrier. The time from death to surgery was one day, and the cornea was stored in Optisol GS. The graft endothelial cell count as measured by a HAI Labs specular microscope preoperatively was 3149 cells/mm^2^. Pachymetry of the donor was 575 microns, from which a 150-micron graft was cut with a microkeratome and further cut with a 9.5 mm trephine.

### 2.4. Surgical Approach for the Recipient

The decision of when and how to proceed with endothelial keratoplasty is a nuanced one that has evolved over time, with approaches to consider before and after surgery. Our protocols for perioperative planning, including anesthesia and postoperative sedation, are included in Supplemental Protocol 3.

Endothelial keratoplasty was initiated, and the patient positioned and prepped using standard operative technique. An anterior chamber infusion was placed through a 1.1 mm perilimbal incisions at 1 o'clock. Through a second 11 o'clock 1.1 mm incision, a reverse Sinskey hook and Terry scraper were directed anteriorly to strip and remove the central 10 mm of Descemet's membrane. We used a 5.2 mm keratome to lengthen the 11 o'clock perilimbal incision and expand it to allow the tip of the inserter to enter the anterior chamber.

A third perilimbal 1.1 mm incision was created at the 5 o'clock position near the limbus. The 23 G microforceps entered the anterior chamber through the ventral 1.1 mm incision and extends out the 11 o'clock incision. The Luna Inserter was turned upside down, so that the endothelial side faced posteriorly. The microforceps grasped the edge of the donor graft. Once purchase of the graft was confirmed, the microforceps directed the graft through the incision and into the anterior chamber. This pull-through method is demonstrated in Video 1, accessible here in the following: https://youtu.be/VYMxF5cg5F0.

We closed the incisions with simple interrupted 10-0 nylon sutures, and a bubble with 20% SF6 gas was placed posterior to the graft to affix it to the cornea. Gas was placed in the anterior chamber via a 27 G needle and 5 cc syringe to create a 65% “fill” of the anterior chamber. The air bubble was left undisturbed for 20 minutes to promote graft attachment. After 20 minutes, a small amount of gas was removed from the anterior chamber, leaving behind a 7-9 mm gas bubble to support the graft after surgery. This bubble is expected to dissolve in about two days but provides structural support until it disappears. 10-0 nylon sutures were used to fix the graft in place using a technique we have reported for human EK [8]. Subconjunctival injections of betamethasone and gentamicin were administered.

Following surgery, intraocular pressure was measured every 4 hours. Topical moxifloxacin, difluprednate, and Optixcare lubricating gel were applied every 6 hours for 1 month, then tapered to twice daily in 3 months, and discontinued at 6 months. Oral medications included amoxicillin/clavulanic acid 12.5 mg/kg BID for 10 days, cyclosporine 5 mg/kg once daily for 30 days, and gabapentin 4 mg/kg BID for 10 days. Prednisolone was tapered from 1 mg/kg BID for 7 days, then 1 mg/kg once daily for 7 days, and then 0.5 mg/kg once daily until the cornea was clear. Dosages are included in Supplemental Protocol 3.

An Elizabethan collar was used 2-3 weeks postoperatively to prevent self-trauma. The positioning of the DSEK graft was evaluated postoperatively with the Accutome UBM.

### 2.5. Intraoperative Outcomes

Surgery was performed as described without complications. Intraoperative fibrin accumulation was not observed, and tPA injections were not necessary intra- or postoperatively.

### 2.6. Postoperative Outcomes

At postoperative day 1, the graft was attached and sutures were intact. Subsequently, the healing course was uneventful, without corneal ulcerations or leakage of SF_6_ gas.

Time from surgery to corneal clearing was approximately 1-2 weeks. At one week, the cornea subjectively began to clear, and pachymetry of the graft and donor combined was 1410 microns. At four months, the cornea was clear and central pachymetry in the operated eye was 800 microns. The patient was seen postoperatively every 6 months, and the graft remained clear at the last visit, 4 years after surgery.

## 3. Discussion

In this case report, we describe our approach and a case of endothelial keratoplasty using a dedicated canine DSEK inserter. The graft was successfully introduced into the eye and resolved the corneal edema without complication.

The data add to previous reports demonstrating feasibility of endothelial keratoplasty in a dog [4, 5]. For monitoring the health of the canine cornea postoperatively, we confirmed improvement through pachymetry, which can be performed in a clinic with an ultrasonic pachymeter. Corneal clarity and decreased corneal thickness were noted as soon as one week.

Endothelial keratoplasty in the setting of canine eyes carries unique challenges, which we have summarized in Table 1. These include a larger graft, a more flexible host cornea that can collapse during surgery, limited visualization through a diffusely edematous cornea, limited ability to position postoperatively, and a robust inflammatory response in canine eyes.

To address challenges with manipulating the large and flexible tissue through the wound, we developed a device to facilitate the insertion of a canine corneal graft. The device was able to maintain the graft's endothelium-in configuration, which facilitates the graft positioning in the anterior chamber and reduces the overall burden on the surgeon. As additional devices are developed, future studies may compare endothelial cell viability across methods of tissue insertion.

We have shared the product files for any collaborators interested in using or improving on the device, accessible at Supplemental File 4. Additional variations to the described inserter may include lumen shape and size, addition of infusion, or removal of the posterior platform.

In both canines and humans, graft detachment is a potential complication with DSEK. In this case, nonabsorbable 10-0 Ethilon sutures were applied dorsally and ventrally to anchor the graft to the adjacent recipient stroma, a technique we have described in humans [8]. However, placement of sutures could lead to other possible complications such as epithelial cell downgrowth or focal fungal or bacterial infection, and sutures should be monitored and/or removed postoperatively.

Although alternative, palliative procedures may be effective in mitigating pain and infections, canine EK can be considered as a first-line approach as it offers partial restoration of vision. Future studies can assess the efficacy and efficiency of this approach in restoring vision and the learning curve for adoption of relevant technologies.

## Figures and Tables

**Table 1:** Characteristics of Descemet's stripping endothelial keratoplasty in human and canine eyes. Surgery in canine eyes carries unique risks and considerations relative to surgery in humans.

	Human	Canine
Diameter of cornea	Approximately 10.5 to 11.5 mm	Approximately 15-16 mm

Pachymetry when presenting for endothelial keratoplasty	Often at 600 microns or beyond [9]	In our practice, an average of 1300 microns

Flexibility of cornea	Cornea usually remains vaulted although chamber may shallow when entering or exiting eye	No Bowman's layer. Cornea is flexible and can collapse with fluid egress from anterior chamber

Ease of removal of Descemet's membrane	Can often be removed as a sheet	Tightly adherent. Can be removed in strips and shreds

Visualization of graft	In most cases, the graft is visible, although orientation markings and trypan staining assist with visualization	Limited. Trypan blue recommended. Complete air bubble and adjustment of lighting allows for visualization of graft edge

Postoperative positioning	Patients generally capable of adherence to positioning requirements. Sutures can assist if needed [8]	Frequent, rapid movements are expected. We use sutures to fix the graft and 20% SF_6_ to provide long-term stability of the gas bubble

Inflammation	Topical steroids	Topical, oral, and sub-Tenon's injections of steroids and anti-inflammatory medication

## Data Availability

The authors have openly shared the files for manufacturing the device in Supplemental File 4.

## Supplementary Materials

Supplementary Materials Video 1: in this pull-through technique using a cartridge designed for the canine eye, the trypan blue-stained, endothelium-in graft is brought to the wound and pulled into the eye using microforceps. Given the limited visualization in the eye, an air bubble can be helpful to see the edges of the graft against the cornea. Supplemental File 1: design requirements and development of canine DSEK inserter. Supplemental File 2: cornea transplant preparation: donor identification, tissue recovery, tissue quality assessment, and tissue cutting. Supplemental File 3: perioperative planning: anesthesia and postoperative sedation for serial assessment. Supplemental File 4: link to design files for canine DSEK inserter.
