# Supplementary material for: Canine Descemet Stripping Endothelial Keratoplasty with a Tissue Insertion Device: Technique and Long-Term Outcome
Source: Case Rep Vet Med. 2023 Dec 21;2023:7497643. doi: 10.1155/2023/7497643 (PMC10754630; doi:10.1155/2023/7497643)
Supplement: Supplementary Materials — Video 1: in this pull-through technique using a cartridge designed for the canine eye, the trypan blue-stained, endothelium-in graft is brought to the wound and pulled into the eye using microforceps. Given the limited visualization in the eye, an air bubble can be helpful to see the edges of the graft against the cornea. Supplemental File 1: design requirements and development of canine DSEK inserter. Supplemental File 2: cornea transplant preparation: donor identification, tissue recovery, tissue quality assessment, and tissue cutting. Supplemental File 3: perioperative planning: anesthesia and postoperative sedation for serial assessment. Supplemental File 4: link to design files for canine DSEK inserter. [file 7497643.f1.zip › 7497643.f1/Supplemental Files 4.docx]

**Supplemental Files 4. Luna Inserter Cartridge and Cap Source Files**

The 3D files and PDF specifications for the Luna Inserter are linked below. Each part is uploaded as a standard triangle language (STL) file format and Solidworks Part (SLDPRT) file format. The STL part can be viewed in standard 3D object viewing software and 3D printed. The SLDPRT can be opened and edited in Solidworks. Please follow the link below and click “Launch workspace” to access the files.

https://data.world/conchen/supplemental-4-luna-inserter-stl-files-and-specifications
