# Supplementary material for: Canine Descemet Stripping Endothelial Keratoplasty with a Tissue Insertion Device: Technique and Long-Term Outcome
Source: Case Rep Vet Med. 2023 Dec 21;2023:7497643. doi: 10.1155/2023/7497643 (PMC10754630; doi:10.1155/2023/7497643)
Supplement: Supplementary Materials — Video 1: in this pull-through technique using a cartridge designed for the canine eye, the trypan blue-stained, endothelium-in graft is brought to the wound and pulled into the eye using microforceps. Given the limited visualization in the eye, an air bubble can be helpful to see the edges of the graft against the cornea. Supplemental File 1: design requirements and development of canine DSEK inserter. Supplemental File 2: cornea transplant preparation: donor identification, tissue recovery, tissue quality assessment, and tissue cutting. Supplemental File 3: perioperative planning: anesthesia and postoperative sedation for serial assessment. Supplemental File 4: link to design files for canine DSEK inserter. [file 7497643.f1.zip › 7497643.f1/Supplemental Protocol 1.docx]

**Supplemental Protocol 1. Design requirements and development of canine DSEK inserter.**

Two specific requirements of the device arose that ensure ease of use and ensure graft viability. First, it must be able to be inserted within a 6 mm corneoscleral incision. Current DSEK technologies in humans use incisions equal to or less than 4.5 mm;^4^ since the canine eye, corneal edema and tissue graft are larger, a larger incision needs to be made, but keeping this incision as small as possible aids in the recovery process. Moreover, as canines do not have a corneal Bowman’s layer, the overall rigidity of the cornea is compromised with a large incision, and can result in anterior chamber collapse intraoperatively. Although cohesive viscoelastics could help expand the anterior chamber, residual viscoelastics would interfere with graft adherence.

Second, it must accommodate the size of the canine corneal tissue: The graft must fit in the device with minimal contact with itself and the inner lumen of the device in order to avoid damage to the cells and friction with the device.

SolidWorks software was used to design the device, and final devices were injection-molded using medical-grade polycarbonate through Xcentric Mold and Engineering (Clinton Twp, MI). Watertight caps were 3D printed using FormLabs 3 (Baltimore, MD).

The device was designed to allow a DSEK graft to be loaded through its distal aspect, and has features to facilitate the delivery and insertion of the graft into the eye during the procedure through an proximal opening. It is also designed to be preloaded before delivery to veterinary ophthalmologists from the graft procurement site.

The main body of the Luna Inserter has a 12.8 mm wide ovular opening that tapers down to a tip width of 4.5 mm. This wide ovular opening at the back end offers a place for the trifolded canine graft to be housed in, while tapering to the tip allows for the device to be inserted into the eye via a corneoscleral incision of 6 mm. Up to a 15 mm graft can be housed in the device.

Central 0.63 mm grooves run along the length of the device, one each on the upper and inner lumen. These provide space for standard 23-gauge micro-forceps to facilitate moving the trifolded graft into and out of the cartridge in addition to preventing the graft from sticking to the device by minimizing surface contact. A watertight back-end cap keeps the trifolded graft secure within the cartridge during transport and prevents the graft from moving backwards out of the cartridge during insertion. An example of this device is illustrated in Figure 1.

The workflow for the device involves several stages. First, the graft is cut from the donor canine cornea. It is then trifolded with the endothelium side inward and pulled into the wide body of the cartridge for loading, and the cartridge filled with medium. If preloaded at the eye bank, the back end cap is attached to the cartridge to secure the graft during transportation to the veterinarian. During the procedure, the surgeon flips the cartridge and inserts the tip of the device into the canine’s eye, using micro-forceps to pull the graft out of the device.

Criteria for a successful insertion device includes adequate protection of the canine endothelial cells, maximal graft survival and maintaining orientation of the graft.
