# Supplementary material for: Canine Descemet Stripping Endothelial Keratoplasty with a Tissue Insertion Device: Technique and Long-Term Outcome
Source: Case Rep Vet Med. 2023 Dec 21;2023:7497643. doi: 10.1155/2023/7497643 (PMC10754630; doi:10.1155/2023/7497643)
Supplement: Supplementary Materials — Video 1: in this pull-through technique using a cartridge designed for the canine eye, the trypan blue-stained, endothelium-in graft is brought to the wound and pulled into the eye using microforceps. Given the limited visualization in the eye, an air bubble can be helpful to see the edges of the graft against the cornea. Supplemental File 1: design requirements and development of canine DSEK inserter. Supplemental File 2: cornea transplant preparation: donor identification, tissue recovery, tissue quality assessment, and tissue cutting. Supplemental File 3: perioperative planning: anesthesia and postoperative sedation for serial assessment. Supplemental File 4: link to design files for canine DSEK inserter. [file 7497643.f1.zip › 7497643.f1/Supplemental Protocol 2.docx]

**Supplemental Protocol 2. Cornea Transplant Preparation: Donor Identification, Tissue Recovery, Tissue Quality Assessment, Tissue Cutting**

**Identification of Donors**

A well-informed and passionate community of pet owners in the Washington DC area who consented to the recovery of their pet's tissue following humane euthanasia for transplantation were identified.

The surgical team's donor eligibility standards for this case were a known cause of death, and exclusion criteria included viral encephalitis, active bacterial or viral meningitis, active bacterial or fungal endocarditis, rabies, and neoplasia. Rabies vaccination status was up to date and traceable through a veterinary establishment. Eyes were excluded with active ocular or intraocular inflammation, glaucoma, or prior ocular surgery. Upon meeting the pre‐recovery tissue suitability criteria, pairs of corneas were recovered by one of the authors (MDA). Final tissue suitability criteria were the following: (a) Cell count greater than 2500 cells/mm^2^, (b) time from euthanasia to preservation (“E to P”) less than 2 hours, and (c) time from euthanasia to transplant (“E to T”) less than 7 days. A complete blood count and serum biochemistry profile performed on the donor patients were within normal limits. A complete ophthalmic examination, including slit lamp biomicroscopy and indirect ophthalmoscopy, was performed on the donor canine patient prior to or directly following humane euthanasia. No malignant ocular tumors were identified. No active intraocular or periocular inflammation was identified. The donor did not have a history of previous ocular diseases or ophthalmic surgeries. Congenital and acquired ophthalmic diseases that would potentially preclude a successful outcome could not be identified.

**Tissue Recovery**

Tissue was recovered from donors as follows. Five minutes following humane euthanasia, the corneas were aseptically prepared with 1:50 povidone iodine solution. Sterile drapes were aseptically placed. A lateral canthotomy was performed and a barraquer eyelid speculum was placed. A peritomy was performed to remove the conjunctiva from the underlying sclera. A 360‐degree circular partial depth incision was created with a number 64 beaver blade 2 mm posterior to the limbus. This incision was followed with westcott tenotomy scissors to puncture into the globe and to free the cornea and 2‐mm scleral rim from the underlying globe. The iris was separated from the cornea. The donor tissue was placed in Optisol GS (Bausch & Lomb) corneal storage media and kept refrigerated at 2‐8°C until tissue preparation began the day of surgery.

**Tissue Quality Assessment**

The donor corneal tissue was placed in a viewing chamber in Optisol solution and closely evaluated for trauma, fibrosis, scarring, and any infiltrates that would decrease cell viability or in any way negatively impact transplantation. The thickness of the cornea was evaluated with ultrasound pachymetry. A specular microscope (Hai Labs) imaged and analyzed the endothelial cells for pleomorphism, polymegathism and to determine the cell density. We only utilized eyes with endothelial cell density of at least 2500 cells/mm^2^.

**Tissue Cutting**

The cornea was placed on a nitrogen-powered microkeratome (Moria Ophthalmic Instruments, Doylestown, Pennsylvania, USA) to perform a lamellar transectional dissection of the anterior segment of the cornea. The maximum diameter attainable from the equipment was 10 mm. The targeted thickness of the post processed graft was less than 150 μm. This very thin section of corneal tissue is created with two separate passes of the microkeratome; the first pass removes the anterior ⅔ of the cornea, and with guidance from a pachymeter, the second pass cut is selected to result in an ideal corneal thickness. The tissue was deemed suitable for transplantation by the authors, was brought into the surgery suite, and was trephined to be 9-9.75mm in diameter endothelium side-up, then manually transferred onto the Luna Inserter. The graft was gently stained with preservative free Trypan blue 0.06% (DORC International, the Netherlands) for visibility purposes.
