# Supplementary material for: Canine Descemet Stripping Endothelial Keratoplasty with a Tissue Insertion Device: Technique and Long-Term Outcome
Source: Case Rep Vet Med. 2023 Dec 21;2023:7497643. doi: 10.1155/2023/7497643 (PMC10754630; doi:10.1155/2023/7497643)
Supplement: Supplementary Materials — Video 1: in this pull-through technique using a cartridge designed for the canine eye, the trypan blue-stained, endothelium-in graft is brought to the wound and pulled into the eye using microforceps. Given the limited visualization in the eye, an air bubble can be helpful to see the edges of the graft against the cornea. Supplemental File 1: design requirements and development of canine DSEK inserter. Supplemental File 2: cornea transplant preparation: donor identification, tissue recovery, tissue quality assessment, and tissue cutting. Supplemental File 3: perioperative planning: anesthesia and postoperative sedation for serial assessment. Supplemental File 4: link to design files for canine DSEK inserter. [file 7497643.f1.zip › 7497643.f1/Supplemental Protocol 3.docx]

**Supplemental Protocol 3. Perioperative planning: Anesthesia and Post-operative sedation for serial assessment.**

**Anesthesia**

Patients were pre-medicated with buprenorphine (0.01 mg/kg), maropitant (1 mg/kg) intramuscularly and famotidine (1 mg/kg) intramuscularly 30 minutes to 2 hours prior to induction. The patients were induced with midazolam (0.2 mg/kg) followed by propofol (4 mg/kg) and placed in dorsal recumbency under an operating microscope. The patients were maintained on 5 mL/kg/h intravenous fluid supportive therapy for the duration of the procedure. Cefazolin (22 mg/kg) and dexamethasone (0.15 mg/kg) were injected intravenously shortly after induction. The patients were maintained on isoflurane inhalant anesthesia for the duration of the surgery.

**Postoperative Care**

Topically, moxifloxacin, difluprednate and Optixcare lubricating gel were applied every 6 hours for 1 month, then tapered to twice daily in 3 months and discontinued at 6 months. Oral medications included Amoxicillin/clavulanic acid 12.5 mg/kg BID for 10 days, cyclosporine 5 mg/kg once daily for 30 days, and gabapentin 4 mg/kg BID for 10 days. Prednisolone was tapered from 1 mg/kg BID for 7 days, then 1 mg/kg once daily for 7 days, then 0.5 mg/kg once daily until corneal vascularization was no longer observed..

An Elizabethan collar was used 2-3 weeks postoperatively to prevent self‐trauma. Intraocular pressures were evaluated every 4 hours postoperatively. The positioning of the DSEK graft was evaluated postoperatively with an Accutome UBM. Patients were sedated with dexmedetomidine and butorphanol 3-5 days postoperatively in order to monitor graft adherence and minimize irregularities that would hinder graft acceptance. The interface between the donor and the recipient graft was monitored as well as for respective corneal thicknesses.
